# Novel Drug Delivery Systems: An Important Direction for Drug Innovation Research and Development

**DOI:** 10.3390/pharmaceutics16050674

**Published:** 2024-05-16

**Authors:** Qian Chen, Zhen Yang, Haoyu Liu, Jingyuan Man, Ayodele Olaolu Oladejo, Sally Ibrahim, Shengyi Wang, Baocheng Hao

**Affiliations:** 1 Key Laboratory of New Animal Drug Project, Gansu Province, Key Laboratory of Veterinary Pharmaceutical Development, Ministry of Agriculture and Rural Affairs, Lanzhou Institute of Husbandry and Pharmaceutical Sciences of Chinese Academy of Agriculture Sciences, Lanzhou 730050, China; 15528168849@163.com (Q.C.); lzmy_yz@163.com (Z.Y.); lhy000503@163.com (H.L.); hk1013264544@163.com (J.M.); oladejoayodele85@gmail.com (A.O.O.); sally_rashad2004@yahoo.com (S.I.); 2 Department of Animal Health Technology, Oyo State College of Agriculture and Technology, Igboora 201003, Nigeria; 3 Department of Animal Reproduction and AI, Veterinary Research Institute, National Research Centre, Dokki 12622, Egypt

**Keywords:** novel drug delivery system, targeting technology, carrier, nanotechnology, three-dimensional printing (3DP) technology

## Abstract

The escalating demand for enhanced therapeutic efficacy and reduced adverse effects in the pharmaceutical domain has catalyzed a new frontier of innovation and research in the field of pharmacy: novel drug delivery systems. These systems are designed to address the limitations of conventional drug administration, such as abbreviated half-life, inadequate targeting, low solubility, and bioavailability. As the disciplines of pharmacy, materials science, and biomedicine continue to advance and converge, the development of efficient and safe drug delivery systems, including biopharmaceutical formulations, has garnered significant attention both domestically and internationally. This article presents an overview of the latest advancements in drug delivery systems, categorized into four primary areas: carrier-based and coupling-based targeted drug delivery systems, intelligent drug delivery systems, and drug delivery devices, based on their main objectives and methodologies. Additionally, it critically analyzes the technological bottlenecks, current research challenges, and future trends in the application of novel drug delivery systems.

## 1. Introduction

DDS represents a promising technological advancement with extensive applications, engineered to release drugs in a controlled manner and at a predetermined rate and deliver them to specific tissues or cell types. Recent drug delivery systems, such as nanoparticles, molecularly imprinted polymers, and 3D printing technology, have emerged as cutting-edge research topics. DDS is a pivotal strategy for achieving targeted and precise drug delivery. Table 1 succinctly introduces the challenges and solutions associated with drug molecular delivery.

By leveraging multidisciplinary approaches, DDS is dedicated to the development of drug delivery systems and devices that can modulate the metabolism, potency, toxicity, immunogenicity, and biorecognition of drugs, thereby enhancing the microenvironment in which the drug operates and facilitating its uptake by the body [1]. Compared to conventional formulations, DDS offers several key advantages: (1) enhanced drug stability and minimized degradation; (2) optimized drug distribution, leading to increased target concentration, and reduced adverse reactions; (3) precise drug localization, timing, and targeted release, such as breaking through the blood–brain barrier for drug delivery and (4) decreased therapeutic dosage, reduced the toxicity, and elevated therapeutic index. DDS not only delivers drugs to the affected area but also encompasses four core functions: drug targeting, controlled release, enhanced drug absorption and improved drug stability. These functions align with the most critical demands in clinical drug applications.

**Figure 1:**
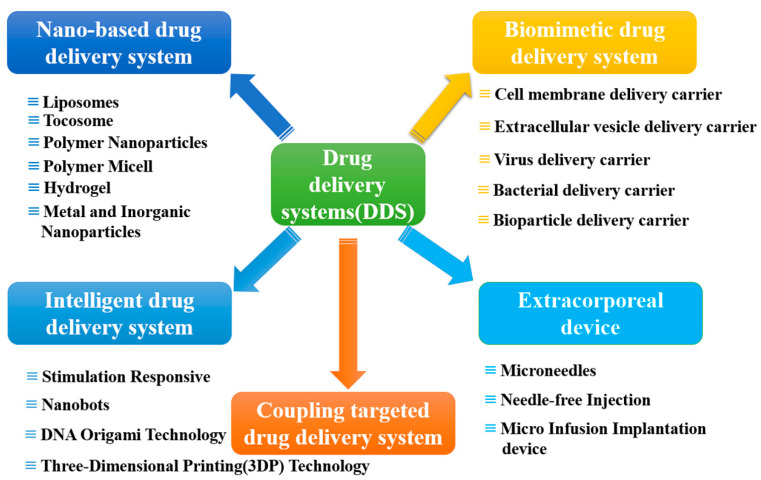
Main types of drug delivery systems.

The research on DDS spans multiple disciplines, intersecting and collaborating with each other. This review presents the latest research advancements in DDS from four perspectives: carrier-based and coupling-based targeted drug delivery systems, intelligent drug delivery systems, and drug delivery devices, aligning with the primary objectives and methods of drug delivery systems (Figure 1). Additionally, the review analyzes and discusses the technological bottlenecks in the application of novel drug delivery systems, as well as the challenges and future development trends of current research.

## 2. Carrier-Based Drug Delivery Systems 

### 2.1. Nano-Based Drug Delivery Systems (NDDSs)

“Nanotechnology” initially proposed in 1959 [2], is currently experiencing rapid scientific and industrial growth, and its integration with biotechnology, information technology and cognitive science has propelled life sciences into a new era. Nanotechnology possesses unique physical, chemical, and biological properties, and the resulting nano formulations are anticipated to find extensive applications in the biomedical field. The application of nanotechnology in constructing drug delivery systems can effectively enhance drug solubility, stability, and tumor targeting, and mitigate toxic side effects [3]. A diverse array of materials is employed in the construction of NDDSs, encompassing liposomes, nanodrugs, polymer micelles, hydrogels, and inorganic nanodrug delivery systems [4,5].

#### 2.1.1. Liposomes 

**Figure 2:**
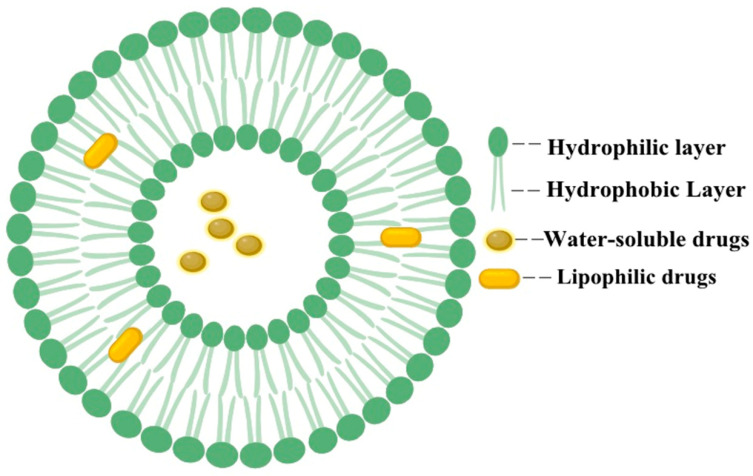
The hydrophilic and hydrophobic structure of liposome drug delivery system.

Liposomes, characterized by their ordered bilayers of lipids forming enclosed vesicles, possess a hydrophobic shell and a hydrophilic core, with a particle size ranging from 20 to 1000 nm [6,7]. Owing to their distinctive composition and structure, liposomes exhibit excellent biocompatibility and can be metabolized normally. Consequently, they can enhance drug solubility and mitigate drug toxicity [8,9,10]. Liposomes are capable of encapsulating both hydrophilic and hydrophobic drugs [11], thereby protecting them from degradation and preventing drug accumulation in other tissues and organs [12] (Figure 2). The development of liposome drug delivery systems grounded in nanotechnology has taken nearly half a century to be integrated into clinical practice; this advancement has catalyzed a quantum leap in the development of anti-tumor, anti-bacterial infection drugs and vaccines. For instance, in the case of the anticancer agent resveratrol, the utilization of solid lipid nanoparticles for its delivery led to a significantly increased brain concentration of resveratrol in Wistar rats compared to free resveratrol, indicating high penetration into brain tumors and minimal systemic toxicity [13]. 

In tumor therapy, liposome-encapsulated radiosensitizers can augment X-ray radiation to tumor sites. Zhao et al. [14] designed an antigen-capturing stapled liposome (ACSL) with a robust structure and bioactive surface, capable of capturing and transporting tumor-associated antigens (TAAs) from lysosomes to the cytoplasm of dendritic cells (DCs), thereby enhancing TAA cross-presentation and inducing a robust T cell-dependent antitumor response and immune memory following local irradiation. Liposomes encapsulating the anticancer agent doxorubicin have notably diminished the cardiotoxicity associated with doxorubicin and reduced the occurrence of adverse reactions, including myelosuppression, alopecia, nausea, and vomiting [15]. Studies have indicated that the anticoccidial activity of decoquinate nanoliposomes (DQNLs), fabricated through thin-film dispersion-ultrasonic methods, was substantially augmented [16]. 

Liposomes are a prevalent strategy for facilitating drug permeation across the blood–brain barrier. Liposomes modified with transferrin have demonstrated efficient drug transport capabilities. In a glioma-bearing mouse model, the therapeutic efficacy of liposomes exhibited minimal systemic toxicity and significant regression of gliomas following non-invasive systemic administration [17]. The combination of lichenin liposomes and rifampicin has been employed in the treatment of multidrug-resistant tuberculosis, markedly enhancing the antibacterial activity of rifampicin [18,19]. The carbohydrate recognition domain (CRD) of the C-type lectin pathogen recognition receptor, DC-SIGN, can be specifically targeted by antifungal liposomes, thereby augmenting the antifungal efficacy of liposomal Amphotericin B (AmB) both in vitro and in vivo [20]. 

Lipid nanoparticles (LNPs) represent a pivotal technology within liposome delivery systems and have emerged as a substantial advancement in the field of oligonucleotide-based therapeutic agents. LNPs are a specialized subset of liposomes devoid of hydrophilic cavities, composed of cationic phospholipids and negatively charged nucleic acid components that are electrostatically complexed, forming multilayer cores interspersed between lipid layers. Oligonucleotides encapsulated within LNPs are safeguarded during delivery, remaining intact and undegraded by enzymes and are effectively delivered to cells, where the contents of the carrier particles are released and translated into therapeutic proteins. The Southwest Medical Center of the University of Texas in the United States has unveiled a groundbreaking strategy called Selective Organ Targeting (SORT). This innovative approach involves the incorporation of SORT molecules into a diverse array of LNPs, enabling the precise targeting of extrahepatic tissues [21,22]. The synergy of SORT in conjunction with various gene editing techniques has significantly advanced the field of gene therapies, particularly those targeting proteins within specific tissues. Shuai et al. [23] have pioneered a novel LNP delivery system (iPLNPs), which incorporates novel phospholipids (iPhos) with enhanced endocytic escape capabilities. By manipulating the chemical structure and proportion of iPhos, organ-selective delivery can be achieved with remarkable precision. In a separate study, Min et al. [24] identified a novel LNP variant featuring an amide bond in its tail, which can be finetuned to target various lung cell types by adjusting its head structure. In 2022, researchers at the University of Pennsylvania administered mRNAs encapsulated within LNPs to mice with heart failure, effectively modifying T cells and restoring their cardiac function. Building upon this success, in 2023, they developed and synthesized ionizable LNPs capable of delivering mRNA to the placenta without crossing into the fetal compartment, potentially offering a new treatment avenue for pregnancy complications such as preeclampsia [25]. At the Fred Hutchinson Cancer Research Center in Seattle, researchers have harnessed the power of gene editing to create chimeric antigen receptor (CAR) T cells from patient-derived T cells [26]. This cutting-edge technology, facilitated by the LNP delivery system, ensures that the encapsulated CAR gene can access the nucleus via nuclear localization, positioning it as an emerging and promising cancer therapy. The modified mRNA-targeted LNPs have demonstrated remarkable potential in reducing fibrosis and restoring cardiac function post-injury, while also generating transient, yet effective CAR T cells in vivo. These CAR T cells hold immense promise as a versatile therapeutic platform for the treatment of a wide array of diseases [27]. In addressing the challenge of exogenous mRNA penetrating the cytoplasm without undergoing degradation by nucleases, COVID-19 mRNA vaccines have universally employed lipid nanoparticles (LNPs) as their delivery vectors. This innovation has significantly advanced the vaccines’ efficacy, stability, and safety profiles [28]. The Nanoprimer technology has been shown to reduce the uptake of LNPs by the reticuloendothelial system (RES), thereby enhancing the bioavailability of LNPs encapsulating human erythropoietin (hEPO) mRNA or factor VII (FVII) siRNA. This results in a substantial increase in hEPO production (by 32%) or FVII silencing (by 49%) [29]. Furthermore, Swingle and colleagues have developed an ionizable lipid specifically for the formulation of LNPs intended for mRNA delivery to placental cells. The leading LNP formulation, encapsulating VEGF-A mRNA, induced placental vasodilation, highlighting the potential of mRNA LNPs as a protein replacement therapy for treating placental disorders during pregnancy [25]. 

Additionally, microbiota transplantation is a pivotal strategy for both the prevention and treatment of diseases. However, the development of oral bacterial therapies is constrained by low bioavailability and inadequate gastrointestinal retention. Lipid membrane-coated bacteria (LCB) represent a straightforward yet highly effective method for encapsulating gut microbes through biointerfacial supramolecular self-assembly. Bacteria encapsulated with additional self-assembled lipid membranes have demonstrated significantly enhanced survival against environmental challenges, with minimal alterations in viability and bioactivity. Moreover, they have improved the therapeutic efficacy of oral administration in two murine models of colitis [30]. Liposomes, as the most extensively studied and successful nanocarriers in clinical applications to date, offer the benefits of low toxicity, excellent biodegradability, non-immunogenicity, and the capacity to safeguard the encapsulated drug from degradation [31]. However, liposomes still have shortcomings, such as low drug loading capacity, poor stability, high production costs, potential toxic side effects, and significant variability in accumulation at tumor sites [32]. The future research and development direction of LNP delivery systems is mainly focused on upgrading targeting and responsiveness to internal and external stimuli (e.g., temperature, ultrasound, enzymes, etc.), so as to achieve precise treatment [33].

#### 2.1.2. Tocosome

Tocosome, a sophisticated colloidal and vesicular bioactive carrier system, predominantly comprises alpha-tocopherol phosphate (TP), a derivative of vitamin E. Vitamin E naturally exists in eight distinct forms, with alpha-tocopherol being the most prevalent, abundant, and biologically active. TP stands out for its narrow particle size distribution, commendable encapsulation efficiency, minimal immunogenicity, exceptional biocompatibility, and augmented dissolution and penetration capabilities, all of which contribute to its prolonged stability [34]. The multifaceted attributes of Tocophersolan render it an adaptable constituent in the engineering of drug delivery systems. Tocosomes, akin to liposomes, are composed of amphiphilic molecules that form bilayer colloidal structures, displaying analogous behaviors in drug delivery mechanisms and release patterns, despite their unique chemical compositions [35].

Clinical research has underscored the myriad health advantages of TP, including its role in atherosclerosis prevention, cardioprotection, anti-inflammatory effects, and inhibition of tumor metastasis [36,37,38]. Alongside TP, Tocopherol formulations incorporate various phospholipid and cholesterol combinations, which have been effectively utilized in the encapsulation and controlled release of the anticancer agent 5-fluorouracil [34].

Sunitinib malate and sorafenib tosylate are both targeted therapies for metastatic kidney cancer, functioning through distinct pathways to impede angiogenesis and tumor proliferation. Fariba and colleagues have pioneered the development of a coated tocosome by blending chitosan (CS) with poly(N-isopropylacrylamide) (PNIPAAm), employing the Mozafari method [39,40]. This temperature-sensitive tocosomal nanocarrier boasts enhanced stability, ideal particle size, and the potential for industrial-scale production, positioning it as a promising and robust drug delivery system for the anticancer drugs sunitinib malate and sorafenib tosylate.

Tocophersolan (TPGS) is a distinctive multidirectional polymer, a polymerized synthetic derivative of vitamin E. TPGS has been sanctioned by the FDA as a secure pharmaceutical excipient. Taxol and docetaxel (DTX) epitomize a category of highly potent, low-toxicity, spectroscopic natural anticancer agents, predominantly utilized in the treatment of ovarian, breast, and bronchial cancers, among others. Their mode of action involves inhibiting cancer cell growth by facilitating microtubule assembly and preventing microtubule disassembly [41]. Qi et al. [42] modified TPGS with cholesterol to create a novel carrier material, TPGS-CHMC, which possesses a lower critical micelle concentration (CMC). TPGS-CHMC diminished mitochondrial membrane potential and cell membrane fluidity in paclitaxel-resistant ovarian cancer cells (A2780/T). In A2780/T tumor-bearing nude mice, TPGS-CHMC/DTX micelles exhibited significantly enhanced antitumor efficacy and reduced toxicity compared to the free DTX solution.

#### 2.1.3. Polymer Nanoparticles 

Polymer nanoparticles (PNPs) are colloidal particles with diameters spanning from 10 to 1000 nm [43]. Liposomes with larger particle sizes are less prone to traverse the endothelial layer or blood–brain barrier, whereas PNPs with smaller particle sizes can readily permeate these barriers to reach the target site. Common PNPs include synthetic polymers such as polylactic acid, poly (lactide-co-glycolide) (PLGA), polyamino acids, and natural polymers like chitosan, alginate, gelatin, and albumin [44]. Research has demonstrated that the PNP drug delivery system is biodegradable, capable of reducing systemic toxicity and irritation, delaying drug degradation, improving drug release kinetics, and enhancing biocompatibility, drug safety, and efficacy [45,46]. Manipulating the degradation/bond scission of polymers can also modulate the in vivo release kinetics and facilitate the clearance of delivery carriers in vivo.

Surface PEGylation of nanomedicines significantly extends their circulation time in the bloodstream and enhances their permeability and retention (EPR) effect. Therefore a Near Infrared (NIR) light-triggered dePEGylation/ligand-presenting strategy has been developed, relying on the thermal decomposition of azo bonds. This approach involves the self-assembly of Dox/Pz-IR nanoparticles from long PEG chain polymers (Pz-IR) connected by thermo-labile azo molecules, cRGD conjugated IR783 (rP-IR) with short PEG chains and doxorubicin. The Dox/Pz-IR nanoparticles achieve an optimal synergistic effect of photothermal chemotherapy at mild temperatures through progressive tumor accumulation, a precisely regulated photothermal effect and NIR-photothermal therapy (PTT) induced pulsated drug release [47]. Van De Ven et al. [48] utilized PLGA as the carrier material and amphotericin B to prepare drug-loaded nanoparticles, demonstrating no significant hemolytic toxicity in vitro and a good safety profile and antifungal effects.

**Figure 3:**
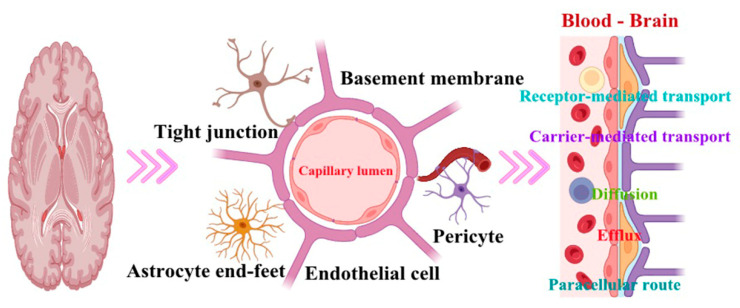
Nanomedicine crossing BBB and delivering therapeutic molecules to target sites in the brain.

Overcoming the regulatory barrier of the blood–brain barrier (BBB) to deliver drugs to the brain remain a significant research challenge. One strategy is the use of nanomedicines capable of crossing the BBB and delivering therapeutic molecules to specific sites in the brain (Figure 3). 

Dendrimers, macromolecules with a dendritic structure formed by repetitive and linear linkage of oligomers via branching units, have shown promise in this regard. Hydroxy polyamidoamine (PAMAM) dendrimers can traverse the BBB and blood-cerebrospinal fluid barriers, effectively delivering small molecule drugs to targeted sites, particularly in injured brain tissue [49]. PAMAM dendrimers with a size of 6.7 nm exhibit longer blood circulation times and greater accumulation in the brain compared to those with a size of 4.3 nm [50]. Furthermore, PAMAM dendrimers with cationic surface properties have been shown to cross the BBB and localize in neurons and glial cells following carotid artery administration [51].

Chitosan, functioning as a distinct receptor on the fungal membrane, has been utilized by Tang et al. [52] to develop chitosan-binding peptide-modified PLGA nanoparticles encapsulating itraconazole. These nanoparticles possess the unique ability to recognize chitosan on fungal surfaces, thereby exerting a pronounced targeting effect on *Cryptococcus neoformans*. Additionally, Chitosan nanoparticles adorned with rhamnolipids (RL) have been loaded with the antimicrobial phytochemical isoliquiritigenin (ISL) (isl@rl-cs). This formulation is capable of concurrently eliminating the biofilm of methicillin-resistant *Staphylococcus aureus* (MRSA) throughout all stages and mitigating the associated inflammation [53].

Researchers at Sloan Kettering Cancer Center have recently unveiled a fucoidan-based nanocarrier that targets endothelial P-selectin, enabling penetration of the blood–brain barrier. Nanoparticles encapsulating the small molecule anti-tumor agent vismodegib were effectively delivered to brain tumor tissues via P-selectin-mediated transport, significantly enhancing the drug’s therapeutic efficacy [54]. 

Molecularly imprinted polymers (MIP), also termed “synthetic antibodies”, are produced through molecular imprinting technology (MIT). The fundamental concept of MIP involves the formation of a template molecule-functional monomer complex through covalent or non-covalent interactions, followed by polymerization in the presence of a cross-linking agent, and ultimately the removal of the template molecule to create a binding site or cavity that matches the template in terms of size, shape, and chemical affinity [55]. Owing to the precise selectivity and affinity of MIP for the template molecule, sustained drug release can be achieved.

Quercetin (3,3,4,5,7-pentahydroxyflavone, QC) is a potent anticancer agent that exerts its antioxidant effects by upregulating endogenous free radical defenses and inhibiting tumorigenesis and tumor progression signaling pathways. However, the clinical application of quercetin for chemoprotection is limited by its hydrophobicity, poor gastrointestinal absorption, and extensive heterologous metabolism in the intestine and liver. A highly selective magnetic molecularly imprinted polymer (MMIP) with a core-shell structure was synthesized by a sol-gel process in the presence of template QC using Tragacanth Gum (TG) crosslinker, Fe_3_O_4_/SiO_2_ nanoparticles, and N-vinyl imidazole (VI) functionalized monomers. The synthesized MMIP nanogel is biocompatible due to the presence of TG, possesses a strong adsorption capacity, is easily separable, and specifically recognizes the template QC [56]. Therefore, MIP and MMIP materials are anticipated to serve as polymeric devices for applications in rapid drug separation and drug delivery.

Polysaccharide nanoparticles (PNPs) have demonstrated significant advancements in the field of drug delivery, with notable achievements in the understanding of their mechanisms of action, environmental interactions, activity profiling, and composite material development. However, the exploration into their potential toxicity, polymer stability, and drug delivery mechanisms remains incomplete [57]. To bridge this gap, future research must delve into a comprehensive and meticulous analysis of the pharmacokinetics, safety profiles, immunogenicity, and other critical aspects of polymer nanodrug delivery systems. This will enable the effective modulation of the physicochemical properties of these systems.

#### 2.1.4. Polymer Micelle 

Polymer micelles, currently in widespread use, are assembled colloidal aggregates formed by amphiphilic block copolymers in an aqueous environment [58]. They are distinguished by their structural integrity, hydrophobic drug solubilization capabilities and minimal toxicity. With a particle size ranging from 10 to 100 nm, these micelles can evade phagocytosis by the reticuloendothelial system, thereby extending their systemic circulation time [59]. Moreover, the hydrophilic shell of the micelles not only prevents drug loss in the serum but also resists complement system activation, which can prematurely quickly clear drugs before they can take effect [60].

In the past three decades, polymer micelles have been extensively employed as carriers for highly potent, highly toxic, and poorly soluble small molecule drugs [61]. Notably, in the realm of antifungal therapy, Albayaty et al. [62] developed an acid-base responsive micellar system for the encapsulation of itraconazole, which boasts a high drug loading capacity and a strong affinity for *Candida albicans* biofilms, significantly inhibiting their activity. Poly micelles also hold the potential to be loaded with combinations of multiple chemotherapeutic agents for targeted tumor delivery, thereby reducing chemotherapy-related adverse reactions and enhancing the survival rate and quality of life for patients with pancreatic cancer. This innovation addresses key challenges in chemotherapy [63]. The micelles developed by Zhang et al. [64], known as Cela/GCTR, possess remarkable characteristics that make them promising candidates for the delivery of hydrophobic anti-tumor agents in the treatment of hepatocellular carcinoma. These micelles exhibit sustained release in the bloodstream and rapid release within tumor microenvironments. The hydrophobic segments are strategically positioned at the core of the nanoparticles to encapsulate hydrophobic drugs, while the hydrophilic segments form the outer corona, maintaining the micelle’s structure in aqueous environments. By attaching specific ligands to the hydrophilic corona, these micelles can traverse the blood–brain barrier via transcytosis and subsequently release their therapeutic cargo upon intracellular disruption. Various block copolymer micelles, including PAA-PEG [65], PLA-PEG, DGL-PEG, PTMC-PEG, and PDSGM-PEG, have been documented to facilitate the transport of therapeutic agents across this barrier. Notably, PLA-PEG micelles loaded with paclitaxel (PTX) and modified with the t-Lyp1 ligand demonstrated enhanced accumulation and internalization in glioma cells, effectively inhibiting tumor progression in animal models [66]. Furthermore, advanced wormlike polymer micelles composed of PEG-grafted poly (2-diisopropyl methacrylate) (PDPA) copolymers (mPEG-b-PDPA) have been engineered to degrade in response to changes in the brain tumor microenvironment, thereby releasing drugs directly into the target tumor [67].

Micelles are also extensively utilized in traditional Chinese medicine preparations, enabling precise control over particle size, encapsulation efficiency, and drug loading for ingredients such as emodin, curcumin, baicalin, and paclitaxel ensuring slow and sustained release. However, due to the minute size of the monomer molecules derived from the extraction and separation processes of traditional Chinese medicine, their current application is largely confined to the synthesis of monomers, with limited research on the direct conversion of traditional Chinese medicine extracts into micelles [68].

Internationally, pharmaceuticals based on polymer micelles have been granted marketing authorization, while domestically, such polymer micelle drugs are still undergoing clinical trials. Despite the existing limitations in clinical application duration and the long-term safety assessment of formulation development, the numerous advantages of polymer micelles are poised to propel their ongoing enhancement and broad application in the delivery of hydrophobic drugs.

#### 2.1.5. Hydrogel 

Hydrogels, which are polymer networks either physically or chemically crosslinked, possess the unique ability to swell in the presence of water and interact with certain organic solvents [69]. The hydrogel nanodrug delivery system exhibits remarkable biocompatibility, biodegradability, and low toxicity, facilitating the sustained release of targeted drugs. 

In the context of tumor therapy, the anti-tumor immune response following radiation therapy is often insufficient, necessitating the use of immune adjuvants to augment the efficacy of antigen-presenting cells. Wang et al. [70] have engineered a hydrogel nanomotor activated by near-infrared light, capable of penetrating tumor tissue and releasing drugs intracellularly, thereby enhancing the immune activation capabilities of the body and achieving a synergistic effect through the integration of phototherapy, chemotherapy, and immunotherapy. Soft hydrogel presents an excellent material option for the repair of various tissue defects. Li et al. [71] have developed an anti-swelling nanofiber hydrogel that boasts high biocompatibility and biodegradability, effectively facilitating fibroblast migration and accelerating angiogenesis during the wound healing process. Sun et al. [72] have developed an innovative hydrogel nanodrug delivery system, which is designed to carry ligands that bind competitively to ATP released from tumor cells upon treatment with oxaliplatin or X-ray irradiation. This system promotes the release of immune adjuvants, thereby enhancing the synergistic therapeutic effect of the treatment.

In the realm of veterinary medicine, Gao et al. [73] have engineered a thermosensitive gel vaccine delivery system that exhibits excellent biocompatibility, degradability, and sustained release capabilities. The Newcastle disease temperature-sensitive gel nucleic acid vaccine, formulated with a recombinant plasmid, has been shown to elicit a robust humoral and cellular immune response, thereby enhancing the body’s antiviral defenses and prolonging the duration of immune protection. Recently, the Shanghai Veterinary Research Institute of the Chinese Academy of Agricultural Sciences has created an innovative supramolecular nanofiber hydrogel (Hydrogel RL) that incorporates antimicrobial peptides. In vitro studies have demonstrated that Hydrogel RL maintains sustained release is biocompatible, and exhibits potent antibacterial activity against methicillin-resistant *Staphylococcus aureus* (MRSA). This development holds promise for combating multidrug-resistant bacteria and addressing healing stagnation resulting from chronic wound infections [74].

The hydrogel system’s resistance to degradation in the gastrointestinal tract allows for sustained drug release. Azad et al. have observed that calcium alginate beads with hydrogel properties remain undegraded in the stomach and are released in the intestinal tract. Moreover, their strong adhesive properties contribute to improved drug retention in the intestinal mucosa [75]. However, the oral hydrogel system has not demonstrated significant progress in clinical trials to date, primarily due to the rapid disintegration of the hydrogel upon contact with substantial intestinal fluids during oral administration. This issue demands focused attention in future research and development endeavors [76]. 

The hydrogel nanodrug delivery system has demonstrated notable advantages in modulating drug release kinetics, enabling the remote and controlled release of drugs, and facilitating the site-specific targeting of drugs. Nevertheless, challenges persist in the clinical application of this technology. Current studies on drug release within hydrogels are largely confined to in vitro experiments or heavily reliant on the internal microenvironment of tumors. Assessing whether hydrogels maintain their response characteristics following in vivo implantation will be a pivotal research focus for hydrogel nanodrug delivery systems moving forward [77]. Moreover, the development of hydrogels necessitates more precise control over the properties of the hydrogel drug delivery carriers and the release kinetics under various trigger conditions. It is evident that highly controllable and precisely adjustable hydrogels possess an expansive application potential in the future [78].

#### 2.1.6. Metal and Inorganic Nanoparticles

In the realm of nanomedicine delivery systems, metal and inorganic nanoparticles, synthesized through physical or chemical methods from metallic or inorganic materials, represent a diverse and promising category. These nanomaterials are distinguished by their exceptional physical and chemical properties, including a high specific surface area, enhanced bioavailability, low toxicity, and compatibility with most organic solvents, Consequently, they have found extensive application in the combined treatment of tumors [79]. Nanographene oxide, carbon nanotubes, mesoporous silica, calcium-based nanomaterials, magnetic nanoparticles, copper nanoparticles, and gold nanoparticles are all significant metallic and inorganic nanocarriers that continue to advance the field of nanomedicine. 

Zhao et al. [80] have integrated copper ions and other substances into oxidative stress amplifiers, thereby sensitizing immunotherapy through chemotherapy. This approach has reversed the immunosuppressive tumor microenvironment, augmented immunotherapy efficacy, and significantly curtailed the growth of primary distal tumors. Their work offers novel insights into the development of combined therapy strategies for inhibiting tumor growth and metastasis. Gong et al. [81] have developed a phosphorus and nitrogen-doped hollow carbon quantum dot DOX carrier, which has been shown to enhance the intranuclear delivery and tumor accumulation of DOX, thereby effectively inhibiting tumor growth. The multifunctional CuS nanocomposite designed for the combined administration of oligonucleotides and docetaxel promotes the infiltration of Tc cells and enhances the therapeutic efficacy of breast cancer when used in conjunction with photothermal and photodynamic therapies [82].

In the field of veterinary medicine, Raposo et al. [83] have prepared and tested the effects of gold nanoparticles loaded with cobalt and zinc compounds on canine cancer cells. Their findings indicate that these nanoparticles are readily surface-modified and more effective in delivering cytotoxic substances than free compounds. Silver has been shown to act on bacterial enzymes and proteins, thereby inhibiting the production of bacterial toxins [84]. Nanosilver nanoparticles (AgNPs) prepared using nanotechnology, not only exhibit the characteristics of nanomaterials but also enhance the antibacterial effect of silver [85]. The potential antibacterial mechanisms of silver nanoparticles (AgNPs) may include disruption of normal bacterial morphology by inhibiting the synthesis of cell wall peptidoglycans and inhibition of bacterial growth by inhibiting the cell division protein FtsZ and the chromosomal replication initiation protein DnaA [86].

Livestock manure serves as a reservoir for a multitude of antibiotic resistance genes (ARGs), and its accumulation of livestock manure on land may foster the emergence of antibiotic-resistant bacteria and facilitate the dissemination of ARGs. Nanoscale zero-valent iron (nZVI), with its expansive surface area and unique physicochemical properties, can effectively reduce the concentration of antibiotics and mitigate the risk of ARG transmission during composting processes [87]. Additionally, copper nanoparticles have demonstrated efficacy in both the prevention and treatment of mastitis [88].

The reactivity of inorganic nanoparticles necessitates surface modification with biocompatible materials to serve as non-invasive nanomedicines. Among these, gold nanoparticles are the most extensively utilized inorganic nanomedicines in biomedical applications due to their ease of synthesis, surface modification capabilities, and high biocompatibility. Research has shown that gold nanoparticles can exploit cleavable bonds within endosomes to facilitate transport across the blood–brain barrier while simultaneously inhibiting blood reflux. 

Rodrigues et al. [89] conjugated transferrin (Tf) and rabies virus glycoprotein (RVG) peptide to the surface of liposomes, targeting transferrin and nicotinic acetylcholine receptors. They characterized the function of these liposomes in traversing the blood–brain barrier using an in vitro triple co-culture BBB model. The liposome RVG-Tf was found to continuously transfect and effectively transport primary neuronal cells in an in vitro blood–brain barrier model, and it was observed to enhance the penetration of the blood–brain barrier in vivo. Wang et al. [90] prepared transferrin-modified liposomes (Tf-PL) for the targeted delivery of acetylcholinesterase (AChE) therapeutic gene to liver cancer cells. These liposomes exhibited higher transfection efficiency than Lipo 2000 and demonstrated a superior targeting effect on liver cancer SMMC-7721 cells in vitro. Furthermore, the subcutaneous injection of Tf-PL/AChE significantly inhibited the growth of liver cancer xenografts in nude mice. Lu et al. [91] have engineered a core-shell nanosphere featuring a liquid phase eutectic gallium-indium core and a thiolated polymer shell. This innovative nanomedicine is a convertible liquid metal system capable of fusing and subsequently degrading under weakly acidic conditions. This mechanism facilitates the release of doxorubicin in acidic endosomes following cellular internalization, thereby demonstrating enhanced chemotherapeutic efficacy in xenograft tumor-bearing mice.

While inorganic nanomaterials can be precisely tailored to meet various drug delivery requirements, their toxicity, biological distribution, and clearance mechanism in vivo are yet to be fully elucidated. To expedite the clinical application of inorganic nanomedicine delivery systems, future research should prioritize the investigation of drug retention effects in vivo and the enhancement of drug clearance processes. 

### 2.2. Biomimetic Drug Delivery Systems

Traditional drug carriers are often plagued by suboptimal biodistribution, abbreviated blood circulation times, and diminished delivery efficiency. Nanostructured drug carriers have the potential to modify the pharmacokinetics and biodistribution of drugs; however, they are prone to recognition as foreign entities by the reticuloendothelial system, which can impede their arrival at intended target sites [92,93]. As nanotechnology, biocoupling, and bioengineering tools continue to advance, researchers are gaining deeper insights into the interactions between natural substances—such as cells and pathogens—and the body’s cellular systems. This understanding has catalyzed efforts to mimic these structures and functions for therapeutic applications [94]. Hence, the study of endogenous carriers with minimal toxicity and robust biocompatibility is of paramount importance. In recent years, biomimetic drug delivery systems utilizing biological carriers such as cells, extracellular vesicles, viruses, and bacteria have emerged as a focal point in the field of drug delivery.

In recent years, biomimetic drug delivery systems based on biological carriers such as cells, extracellular vesicles, viruses, and bacteria have become a research hotspot in the field of drug delivery [95]. The biological carrier inherits the structure and function of the original donor, functioning as an endogenous substance to mitigate unnecessary immune responses and evade direct elimination by monocytes and macrophages. Moreover, these carriers can also mimic the structure of highly infectious agents or pathogens within the body, replicating their internal processes or mechanisms of action, and ensuring the precise delivery of medication to the target site for optimal therapeutic effect. Consequently, biological carriers are esteemed as a highly promising system for targeted drug delivery [96]. 

#### 2.2.1. Cell Membrane Delivery Carrier

The cell membrane delivery carrier represents a swiftly evolving, multifaceted drug delivery system. It maintains a membrane structure akin to somatic cells, offering superior biocompatibility and minimal toxicity, which confers it unparalleled advantages over other drug delivery vehicles [97]. Cell-based drug delivery systems can be readily fabricated with minimal loss of membrane proteins and intact membrane structures, thereby endowing the carrier with a diverse array of biological functions and target specificity [98]. Owing to its distinctive attributes, such as prolonged circulation time, adaptable morphology, low immunogenicity, and precise targeting, it is increasingly recognized as an ideal drug delivery carrier [99]. Biomimetic nanosystems derived from various natural cells and hybrid cell membranes have demonstrated their efficacy in effectively managing targeted drug delivery systems. These systems can reduce the immune system’s clearance rate, extend blood circulation time, enhance drug loading and targeting, and thereby amplify the therapeutic efficacy against tumors [100].

Currently, the primary carriers employed for cell delivery encompass red blood cells, platelets, various white blood cells, stem cells and cancer cells. Among them, the red blood cell drug delivery system, with its abundant raw materials and strong targeting ability, has achieved remarkable results in the treatment of various diseases through its further development and expansion [101]. Cao’s team has developed cell membrane-coated bacteria (CMCB) utilizing red blood cell membranes and its potential as a powerful tumor imaging agent has been demonstrated through evaluation results in various mouse models [102]. Platelets accumulate at the wound site following surgical resection, causing inflammation of the tumor microenvironment and playing a repairing role. Wang et al. [103] coupled anti-PDL1 monoclonal antibodies to the surface of platelets, effectively releasing anti-PDL1 through platelet-derived particles during platelet activation, thereby reducing tumor recurrence and metastasis in the postoperative period. 

The nanoparticles coated on T cell membranes contain T cell surface antigens crucial for the binding of the human immunodeficiency virus HIV, which can simulate host cell functions to neutralize the virus. As a novel therapeutic agent against HIV infection, they have shown great potential [104]. In recent years, chimeric antigen receptor (CAR)–T cells have emerged in various DDS due to their ability to modify patients’ own T cells to recognize tumor antigens and activate local cytotoxic immune responses. Ma et al. [105] found that CAR-T cells can specifically recognize tumor-associated antigens, and CAR-T membrane-encapsulated NPs can be used for the highly specific treatment of liver cancer. Although CAR-T cell therapy has been successful in clinical practice, issues such as long duration of treatment and high cost have limited its development for the treatment of B-cell malignancies. In their groundbreaking work, Agarwalla et al. [106] have delineated an implantable Multifunctional Alginate Scaffold (MASTER) designed for T-cell engineering and release. This innovative scaffold facilitates the swift generation of CAR-T cells, enabling their deployment into the bloodstream to regulate the proliferation of remote tumors. Such a development promises to streamline the delivery of these therapies, thereby mitigating the complexities and resource demands typically associated with their administration. Presently, CAR-T cell therapy is primarily indicated for B-cell carcinoma, although its efficacy may be compromised by a multitude of factors. To address this, investigators at the Memorial Sloan Kettering Cancer Center have engineered a novel CAR-T cell variant, termed synthetic enzyme-armed killer (SEAKER) cells. These cells, upon engagement with tumor cells, demonstrate augmented anticancer efficacy in vitro and in vivo settings when coupled with small-molecule prodrugs [107]. 

The cell membranes of macrophages and other phagocytic cells are known to possess pattern-recognition receptors that can identify and recognize pathogens and serve as natural ligands for targeted drug delivery. Li et al. [108] have enveloped collagen-based nanoparticles with macrophage membranes, thereby enhancing biocompatibility, amplifying nanoparticle accumulation at sites of infection, and bolstering antibacterial efficacy. Tumor cell membranes, with their inherent tumor-targeting capabilities, have been exploited by Guo et al. [109] to create biomimetic nanoparticles (gct@cm NPs) that encapsulate tumor cell membranes, thereby achieving tumor-specific targeting. Harris et al. have demonstrated that nanoparticles coated in cancer cell membranes (CCM) exhibit a dual mechanism of shielding and targeting, with the modified drug delivery system being preferentially internalized by tumor cells while minimizing uptake by liver cells [110].

Huang et al. investigated the potential clinical application value of different cell membrane-encapsulated nanocarriers for the targeted delivery siRNA. This approach has demonstrated efficacy when compared to exosomes and other delivery systems. Notably, biomimetic cell membrane-coating nanotechnology emerges as a promising strategy for targeted siRNA delivery in cancer therapy [111].

**Figure 4:**
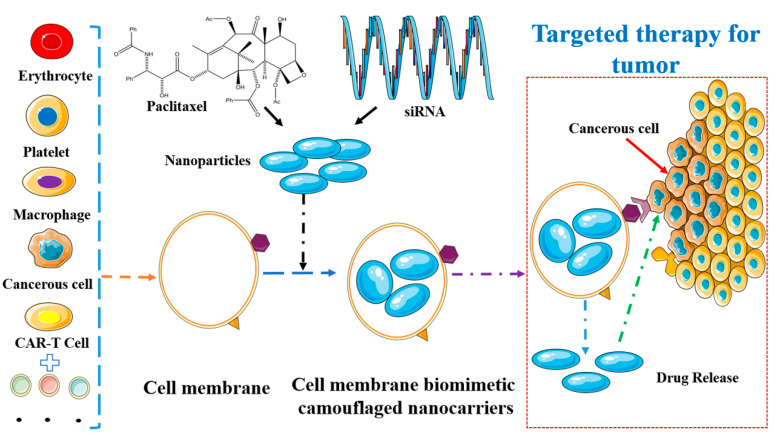
Cellular biomimetic drug delivery system integrating natural cell membrane functions and nanocarrier functions.

The integration of natural cell membrane functions with nanocarrier properties in a cellular biomimetic drug delivery system offers a promising avenue for diverse applications (Figure 4). These cell-derived membrane biomimetic nanocarriers exhibit prolonged circulation times, excellent biocompatibility, and robust immune evasion capabilities. However, the current research is still in its nascent stages, necessitating further investigation into the toxicity, biodistribution, and immune responses of these nanocarriers. Despite this, the inherent benefits of nanocarriers and the abundant availability of cell membranes hold significant potential for therapeutic advancements [112].

#### 2.2.2. Extracellular Vesicle Delivery Carrier

In the realm of extracellular vesicle (EV) delivery systems, these small vesicles released by cells contain biologically active molecules such as proteins and miRNAs. EVs serve as biocompatible carriers with inherent material transport properties, low immunogenicity, and no cytotoxic or mutagenic effects. They possess favorable circulatory stability, biocompatibility, physicochemical stability, and the ability to traverse biological barriers [113]. Specifically, macrophage-derived EVs are capable of penetrating the blood–brain barrier, interacting with cancer cells, and accumulating within them [114]. 

Exosomes, a type of extracellular vesicles, have been the subject researches of extensive research since 2013, particularly those with diameters ranging from 40 to 100 nanometers. These nanoscale vesicles, secreted by the majority of cells, exhibit inherent stability, biocompatibility, minimal immunogenicity and low toxicity. Their unique ability to target specific cells makes them ideal biological nanocarriers for biomedical applications [105]. Moreover, exosomes are preferentially enriched in tumor tissues compared to normal tissues. By conjugating tumor-targeting ligands to exosomes, specific and targeted delivery can be achieved [115,116,117], facilitating the delivery of proteins, peptides, nucleic acids, and other compounds via various routes such as intravenous, intraperitoneal, oral, and intranasal administration. Tumor-derived exosomes, when employed as carriers, can effectively target cancer cells, safeguarding therapeutic compounds from degradation by the extracellular environment, while also maintaining their biocompatibility and low immunogenicity [118]. Exosomes secreted by dendritic cells are rich in antigen-presenting and costimulatory molecules, capable of activating T cells, enhancing the function of natural killer cells, and promoting tumor eradication [119]. Exosomes derived from immune cells possess immunomodulatory properties and therapeutic potential, expressing various antigens on their surface that are instrumental in antigen presentation, immune activation, and metabolic regulation for cancer cell elimination, thereby playing a significant role in cancer therapy [120]. Recent studies have demonstrated that custom-engineered exosomes can be produced by implanting cells into the body [121]. For instance, engineered exosomes produced by implanting cells into live mice have been shown to sustainably deliver mRNA to the brain for the treatment of Parkinson’s disease, opening up a novel avenue for the in vivo production of engineered exosomes [122]. 

Engineering exosomes have significantly enhanced the efficacy and precision of therapeutic agent delivery, making them integral to targeted therapeutic research across various diseases, including oncology, inflammatory conditions, and degenerative disorders. These engineered exosomes possess multifaceted functions such as therapeutic agent loading, target modification, evasion of mononuclear phagocytic system (MPS) phagocytosis, intelligent control, and bioimaging, positioning them as cutting-edge multifunctional nano-delivery systems [123].

In contrast to synthetic nanocarriers, extracellular vesicle drug delivery systems exhibit substantial advantages in terms of targeting, safety, and pharmacokinetics. Nonetheless challenges in extraction, low separation efficiency, high heterogeneity, limited targeting capabilities, and reduced intracellular drug efficacy currently hinder the clinical application of extracellular vesicles [124,125]. Despite being in the nascent stages of research, exosomes hold immense promise as diagnostic biomarkers and anti-tumor drug carriers. Moreover, artificial extracellular vesicles or extracellular vesicle mimics have emerged as a focal point in the field of extracellular vesicle drug delivery, given their advantages of sterility, mass-producibility, and ease of regulation [126,127].

#### 2.2.3. Virus Delivery Carrier

Virus nanoparticles (VNP), derived from bacteriophages and animal and plant viruses, represent a novel class of nanoparticle carriers. These VNP, ranging in size from 10 to 1000 nm, include some infectious varieties. The inherent ability of viruses to infect cells has highlighted their potential as delivery vectors. The first instance of drug delivery utilizing viruses as carriers was realized in 1977. Viral vectors are extensively employed in both in vivo and in vitro in vivo and in vitro drug delivery research, largely due to their exceptional efficiency in gene delivery and expression [128].

At present, the primary virus vectors encompass three main categories: lentivirus (LV), adenovirus (ADV) and adeno-associated virus (AAV). The most extensive application domain is within gene therapy, where 70–80% of gene therapy programs are executed through virus vectors. The cutting-edge gene editing technology, CRISPR, holds significant promise in the treatment of congenital diseases and tumors [129]. In 2014, Cheng et al. developed an in vivo gene editing-based adenovirus CRISPR/Cas9 system, which should achieve a tissue-specific gene knockout level, resulting in phenotypic changes [130]. 

To enhance the stability, cellular targeting, and therapeutic efficacy of CRISPR drug delivery systems, both viral and non-viral vectors can be utilized concurrently to amalgamate the benefits of both vector types. For instance, in 2016, Yin et al. [131] employed AAV vectors to deliver sgRNA, while liposome materials were used to deliver Cas9 protease RNA. The combined deliver of the two vectors to a liver injury mouse model mitigated the symptoms of liver damage and lowered the CRISPR off-target rate. 

With the swift advancement of biological sequencing technology, researchers have recognized the diversity of protein family sequences. Machine learning (ML) models trained directly on experimental data provide a means to harness the full potential diversity of engineered proteins. Bryant et al. [132] applied deep learning to design variants of adeno-associated virus 2 (AAV2) capsid proteins that can effectively load DNA, which has vast potential applications in generating improved viral vectors and protein therapies. Fibroblast activating protein (FAP) expression is detected in the tumor stroma in over 90% of cancers, making it an optimal target site for tumor-specific adenovirus delivery. A sophisticated gene therapy platform, termed SHREAD (Shielded, Retargeted Adenovirus), has been meticulously engineered to selectively target cells based on specific surface markers, effectively converting them into biofactories capable of secreting therapeutic molecules [133]. Human adenovirus serotype 5 (Ad5), a prevalent viral vector, was demonstrated by Hartmann et al. to be retargeted to FAP+ fibroblasts both in vitro and in vivo settings. This approach facilitated the efficient delivery and in situ release of therapeutic agents within the tumor microenvironment (TME), thereby significantly inhibiting tumor growth [134].

As their name implies, oncolytic viruses (OVs) possess the unique ability to dissolve tumors. Wu et al. [135] developed a novel viral strategy for covert tumor targeting, where OVs were treated with liquid nitrogen shock to eliminate pathogenicity, achieving targeted tumor delivery and preventing viral clearance in the bloodstream.

While viral vectors offer high transfection efficiency, they are not devoid of safety concerns and limited loading capacity, which can be limiting for large-scale production. Strategies such as the removal of non-essential viral genes to mitigate toxicity or the construction of self-inactivating viral vectors can enhance the safety profile of these vectors [136].

#### 2.2.4. Bacterial Delivery Carrier

The fusion of chemical biotechnology with bacterial systems has paved the way for bacterial-based drug delivery systems. Engineered bacteria, for instance, are extensively utilized for targeted drug delivery due to their remarkable capability to detect changes in host physiological and pathological indicators, coupled with their superior in vivo transport capabilities [137,138,139]. Some bacteria exhibit a propensity towards hypoxic microenvironments, enabling them to deliver drugs to such challenging environments [140,141,142]. In addition to the noted interactions, there are specific associations between fungi and bacteria [143] such as, *Streptococcus* adhering to *Candida albicans* through cell surface polysaccharide receptors and peptide adhesins and peptide adhesins. Similarly, *Lactobacillus acidophilus* and *Lactobacillus salivarius* can recognize and coaggregate with *Candida albicans* through polysaccharide receptors, offering insights for the development of targeted antifungal drugs [144,145,146,147].

Solomon et al. have engineered a delivery system encapsulates paclitaxel with bacterial vesicles, capable of targeting the overexpressed epidermal growth factor receptor (EGFR) in solid tumor cells, thereby exerting potent anti-tumor effects in xenograft models. The challenge lies in maintaining the control over the physicochemical properties of nanoparticles while emulating bacterial immunogenic traits to activate the immune system. In this context, the bacterial outer membrane emerges as an appealing immune stimulant that, when integrated with nanoparticles, can fine-tune their physical and chemical attributes, giving rise to bacterial membrane coated nanoparticles (BM-NP) [148]. Gao et al. [149] used *Escherichia coli* as a model organism to coat nanoparticles with the outer bacterial membrane, creating BM-NP. These BM-NP demonstrated, through in in vivo experiments in mice, a significantly enhanced capacity to activate dendritic cells, stimulate antibody production, and induce T cell responses against *E. coli* infection compared to the use of the bacterial outer membrane alone. Wang et al. [150] induced the secretion of extracellular matrix Formby bacteria to form a natural biofilm, encapsulating probiotics, which significantly improved gastrointestinal tolerance and mucosal adhesion in both mice and pigs. Futhermore, in mice colonized with *Staphylococcus aureus*, this approach resulted in significantly enhanced decolonization effect.

Bacteria-based DSS have demonstrated considerable advancements in both research and clinical trials. However, they continue to encounter challenges in practical clinical applications, including the scaling up of production, enhancing bacterial survival during drug delivery, precise control of bacterial colonization, dosage determination, and potential biosafety issues. Researchers are committed to bring about in biomedical science by broadening the scope of application in this field through biological and chemical engineering strategies [139].

#### 2.2.5. Bioparticle Delivery Carrier

Various biological particles, such as virus-like particles (VLPs), have gained significant attention as effective carriers for RNA delivery. The viral structural proteins that form the viral capsid can naturally interact with RNA packaging signals (PS) and facilitate the transfer of RNA between cells in the form of VLP. However, the binding specificity of this capsid protein from retroviruses (such as HIV-1) is not particularly strong, and it can also package other RNA molecules. The selectivity of VLPs for RNA molecules can be improved by fusing RNA binding proteins or incorporating specific recognition sequences into RNA molecules. It is important to note that this approach may potentially interfere with the assembly or secretion process of VLP.

Segel et al. [151] identified an endogenous protein, PEG10, capable of forming VLPs from human cells and preferentially binds and promoting the vesicular secretion of its mRNA, selectively encapsulating and delivering other RNAs. Utilizing human PEG10, they developed a system for packaging, secreting, and delivering specific RNAs, termed selective endogenous cell delivery encapsulation (SEND). Derived from human viruses, this system elicits a smaller immune response than existing virus vectors and lipid nanoparticles, and it can efficiently deliver gene editing tools into mouse and human cells to achieve the editing of target genes. The eVLP developed by Banskota et al. has demonstrated its proficiency in mediating highly effective base editing in human and mouse cells, as well as in various tissues and organs of mice, with exceptionally low off-target rates. This innovative system effectively integrates the key benefits of both viral and nonviral delivery methods, positioning it as a promising candidate for therapeutic macromolecular delivery [152].

Endosymbiotic bacteria, a specialized class of microorganisms capable of parasitizing host cells and secreting biological factors that regulate host cells, have evolved intricate delivery systems. One such system is the extracellular contraction injection systems (eCISs), a syringe-like macromolecular complexes that inject carried proteins into eukaryotic cells. Kreitz et al. [153] chose an eCIS from Photorhabdus asymbiotica (Photorhabdus virulence cassette, PVC) for their research. The PVC system has the potential to be reprogrammed for delivering various proteins to human and mouse cells, holding significant promise for applications in gene therapy, nucleic acid delivery and biological control.

The biomimetic drug delivery system represents an advanced integration of biological carriers and functional agents, inheriting the superior properties of natural carriers. Following modification, these systems also exhibit improved permeability, carrying capacity, and specificity [154]. Currently, the biomimetic drug delivery system faces critical challenges, including the elucidation of its in vivo mechanism of action, in vitro modification techniques, and the impact of drug loading on the system itself. In addition, the separation and purification of cell membranes remain an issue that demands attention [155]. Among the promising biomimetic drug delivery systems, mixed cell membrane stands out for its prolonged circulation time and active localization properties [156]. As research progresses, biomimetic drug delivery systems are poised to become ideal candidates for efficient and targeted drug delivery systems.

## 3. Coupling Targeted Drug Delivery Systems

**Figure 5:**
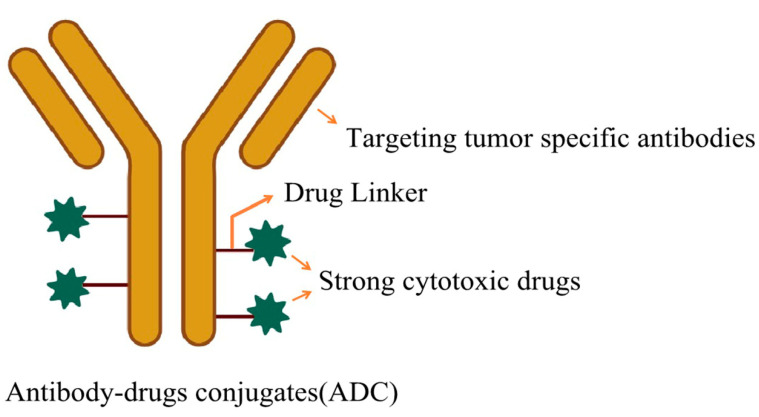
The coupled targeted delivery technology represented by antibody–drug conjugates (ADC).

In recent years, the field of drug delivery has witnessed remarkable advancements, particularly in the realm of targeted delivery technologies. Among these, antibody–drug conjugates (ADCs) have emerged as a cutting-edge approach, where a targeting molecule is chemically bonded to a drug molecule, creating a sophisticated therapeutic agent capable of self-directed delivery to tumor sites upon successful development. An ADC comprises three essential components: antibodies, linker chains, and drugs. The antibodies serve as the delivery vehicle, binding with high specificity to tumor-associated antigens, thereby directing the drug to the intended site. When used in conjunction with conventional chemotherapy, ADCs have demonstrated enhanced efficacy compared to traditional chemotherapy alone, holding immense promise for targeted therapies (Figure 5). To date, the U.S. Food and Drug Administration (FDA) has approved 13 types of ADCs for the treatment of various blood and solid organ cancers (Table 2) [157]. CD276 is a promising cancer treatment target, and Feng et al. [158] referenced the literature to develop a fully human CD276 monoclonal antibody–drug conjugate that significantly improved the therapeutic index and provides an advanced ADC development platform for effective and selective targeting of various types of solid tumors. Xu et al. [159] have developed a novel ADC targeting trophoblast cell-surface antigen 2 (TROP2) for the treatment of TROP2-positive pancreatic cancer.

In the 1960s to 1970s, scientific research revealed that lactose could bind to the asialoglycoprotein receptor (ASGP-R) on the liver’s surface, leading to its internalization. Among lactose analogs, GalNAc (N-acetylgalactosamine) exhibits the most potent binding affinity. GalNac (N-acetylated galactosamine) conjugation has since become the most prevalent small nucleic acid drug delivery system, following its pioneering use by Alnylam Pharmaceuticals. In recent studies conducted by Alnylam researchers, it has been discovered that O-hexadecyl (C16) modified siRNA can effectively penetrate the central nervous system, eyes, or lungs and produce gene knockout effects for a duration of up to three months [160]. This innovative modification represents a departure from the conventional GalNac conjugation and signifies a significant advancement in overcoming the limitations of liver-targeted drugs.

Wang et al. have reported the development of an Active Tissue Targeting via Anchored Click Chemistry (ATTACK) technique, which enables the selective labeling of cancer cells in vitro [161]. This method involves the modification and labeling of the cell surface with a glycoside-containing azide (Ac4ManNAz), resulting in a significantly higher surface azide content in tumor cells due to their heightened affinity for sugars. The resulting conjugates possess the capability to recognize azides and irreversibly target tumor cells, facilitating the release of toxins or other therapeutic agents through click chemistry. Shaqi biopharmaceuticals has developed the Click Activated Protodrugs Against Cancer (CAPAC™) platform, SQ3370, which comprises a tumor-localizing biopolymer and a doxorubicin protodrug. SQ3370 has achieved controlled, tumor-specific drug release, with the potential for clinical application [162].

The Peptide-drug conjugate (PDC) integrates the advantages of peptides, featuring a small molecular weight, biodegradability, and a lack of immunogenic reactions. By modifying the amino acid sequence of the peptide chain, the conjugated hydrophobicity and ionization of PDC can be changed, thereby addressing issues of poor water solubility and untimely metabolism. This modification also enhances the permeability of cells and tissues, overcoming the difficulty of clinical development of small molecule drugs due to poor physicochemical properties. Some specific peptide carriers can also surmount tumor resistance and achieve drug delivery across the blood–brain barrier. Moreover, compared to ADC technology, PDCs offer various industrial benefits in comparison to ADC technology, including improved uniformity, reduced production costs, and shorter cycle times. It can significantly improve the circulation stability and targeting of drugs and has been extensively employed in drug delivery. Gong et al. developed a PDC drug, OPDC3, which can target cells with high peptidase activity and is expected to be a novel drug for the treatment of malignant tumors in various blood systems [163].

In addition to ADC and PDC drugs, there are a variety of clinically available conjugated drugs that achieve delivery functions, including antibody cell conjugated drugs (ACC), viral drug conjugates (VDC), antibody fragment conjugated drugs (FDC), antibody oligonucleotide conjugates (AOC), antibody immunostimulatory conjugates (ISAC), antibody biopolymer conjugates (ABC), etc. 

## 4. Intelligent Drug Delivery System

### 4.1. Stimulation Responsive 

Stimulus-responsive drug delivery systems refer to drugs that are not released or are released extremely slowly until they reach the target tissue or organ, and are released in an adjustable or programmable manner once they reach the target location. With the advancement of synthetic biology, genetic engineering, and photogenetics technology, intelligent cell factories based on small molecules and light signal responses are gradually becoming feasible. Stimulus-responsive drug delivery systems utilize endogenous triggering factors such as pH, reactive oxygen species, and enzyme content, as well as exogenous triggering factors such as temperature, light, magnetic field, ultrasound, electric pulse, and high-energy radiation to trigger or enhance drug release, and control the drug release to be turned on and off on demand via remote devices [164,165,166,167]. 

The stomach’s secretion of gastric acid establishes a distinct pH environment within the stomach, which is notably different from that of other digestive tissues. Leveraging this unique characteristic, researchers have engineered a pH-sensitive intelligent drug delivery system. Feng and colleagues enhanced the gut microbiota’s surface with an intestinal nanocoating, thereby prompting the reactivation of the gut flora [168]. This coating ensures that the bacteria remain dormant upon oral administration, thereby preventing damage from the acidic stomach environment. Following gastric emptying, the subsequent decrease in pH serves as a catalyst for the revival of bacterial activity. This on-demand reactivation of bacteria offers a robust platform for the advancement of highly precise bacterial-mediated biological agents. Similarly, the activation of thermosensitive drug delivery systems can enhance the efficacy of drug release, addressing the issue of excessive heat that can impede drug delivery in various pathological conditions, such as tumor sites and inflammation [169]. Cheng et al. prepared PMEECL-b-POCTCL dual block copolymer with a critical dissolution temperature of 38 °C [170]. Above this temperature threshold, the polymer micelles dissolve, releasing Nile Red and doxorubicin. Additionally, Celsion is developing a heat-activated liposome encapsulation agent for doxorubicin, which is intended for the treatment of various cancers, including breast cancer. 

Light stands as the most optimal external stimulus for intelligent drug delivery systems. Chromophores such as azobenzene, spiropyran, succinic acid, and triphenylmethane can confer photosensitivity to these systems. Indocyanine green (ICG) is an excellent near-infrared dye, and Huang’s research demonstrated that nanomaterials loaded with novel indocyanine green offer advantages in controlled drug release, improved drug stability, targeted delivery, and enhanced drug utilization [171]. Jin et al. have developed a photothermal/reactive oxygen species dual-response biodegradable drug delivery system that responds to both photothermal and reactive oxygen species stimuli, designed for the synergistic treatment of hepatocellular carcinoma with 880 nm laser and reactive oxygen species. This system enables the on-demand release of therapeutic agents directly within the tumor microenvironment, thereby enhancing the efficacy of photothermal and photodynamic chemotherapy treatments [172]. 

Magnetic stimulation represents a non-invasive therapeutic approach. The unique magnetic properties of Fe_3_O_4_ nanoparticles can be harnessed in conjunction with nanotechnology to develop highly precise magnetic-sensitive nanodelivery systems. These systems facilitate the targeted delivery of magnetic nanoparticle-conjugated drugs [173,174]. Hosseini et al. [175] have synthesized superparamagnetic iron oxide nanoparticles for the delivery of anti-cancer therapeutics, demonstrating the potential of this approach.

Ultrasound, known for its high safety, excellent tissue permeability, and cavitation effect, can significantly enhance drug delivery when used in conjunction with microbubbles. The thermal and mechanical effects induced by ultrasound can transiently elevate vascular permeability and destabilize drug-loaded nanoparticles, thereby triggering drug release and amplifying therapeutic efficacy in diseased tissues. Zhu et al. [176] encapsulated paclitaxel in lipid microbubble shells and did not observe significant liver and kidney damage when it was applied in animal models. Ultrasound examination results showed that it can effectively identify the restenosis area of the iliac artery and effectively inhibit neointimal proliferation at the injury site. Paris et al. [177] have developed an innovative ultrasound-responsive drug delivery system based on mesoporous silica nanoparticles, which released doxorubicin from its nanopores under ultrasound irradiation. 

In addition, Yin et al. have developed an engineered cell whose activity is modulated by protocatechuic acid (PCA), an abundant compound [178]. The results indicate that PCA can effectively regulate RNA expression, control the CRISPR-Cas9 system, and modulate insulin release, showcasing the potential of PCA as a regulatory switch in therapeutic applications.

### 4.2. Nanobots

Nanorobots represent sophisticated artificial intelligence systems designed to mimic the intricate biological nanomachines involved in vital life processes and significant biological events [179]. These nanomachines are capable of autonomously navigating their environment and interacting with cells or tissues by harnessing various forms of ambient energy [180]. With dimensions ranging from 1 to 100 nanometers, nanorobots are particularly suitable for targeted drug delivery within the human circulatory system and for minimally invasive diagnostic and therapeutic interventions, thanks to their minute size and capacity to navigate through complex and narrow human tissue networks [181,182,183].

The DNA octahedral framework manufactured by Wang et al. [184] has demonstrated the capability to transport precise quantities of monovalent nucleic acid aptamer-drug conjugates (CA4-FS) directly into solid tumors, facilitating the release of drugs and achieving highly targeted delivery of chemical therapeutics. Xu et al. [185] ingeniously engineered a nanorobot using myocardial cells. Yasa et al. [186] have created a composite algae nanorobot that can maintain movement and survival capabilities in biological media. An orally administered robotic drug delivery capsule known as RoboCap has been developed to locally clear the mucus layer, enhance luminal mixing, and deposit the drug payload in the small intestine, thereby significantly enhancing drug absorption. Studies have indicated that the administration of vancomycin (a 1.4 kDalton glycopeptide) and insulin (a 5.8 kDalton peptide) via RoboCap increased bioavailability by 20 to 40 times (*p* < 0.05) compared to conventional oral administration, both in vitro and in vivo models [187]. 

VLPs require multiple injections for the treatment of abdominal metastases due to the expansive peritoneal space and rapid excretion rates. Scientists at the University of California have harnessed the power of hydrogen, produced through the reaction of magnesium metal with acidic solutions, to propel the targeting delivery of VLPs to tumor tissue. This innovative approach offers fresh insights into the development of tumor immunotherapy strategies for various malignancies within the peritoneal cavity.

The application of targeted drug delivery nanorobot technology holds the promise of delivering therapeutic agents directly to diseased tissues, thereby minimizing adverse effects on healthy organs and tissues. This precision significantly enhances treatment efficacy, reduces the duration of therapy, and cuts down on healthcare costs, thereby facilitating its integration into clinical practice [188,189].

### 4.3. DNA Origami Technology

In the realm of DNA origami technology, scientists exploit the unique properties of DNA molecules and the principles of base pairing to fold long strands of DNA into intricate structures, which are then stabilized by shorter DNA strands. This technology has witnessed remarkable advancements in the assembly of complex structures.

This technology has witnessed remarkable advancements in the assembly of complex structures. However, the surface charge of DNA can impede the deposition of other components, a challenge addressed through the collaboration between Fan Chunhai and Hao Yan, who developed DNA-SiO_2_ composite materials [190]. Building on this foundation, the National Center for Nanoscience and Technology in China has engineered a DNA nanorobot for intravenous administration. This nanorobot is capable of specifically delivering thrombin to tumor-associated blood vessels, triggering intravascular thrombosis and subsequent tumor cell death, thereby effectively inhibiting tumor growth [179].

The rapid advancement of DNA structural nanotechnology has unveiled DNA origami as a promising platform for drug delivery, offering expansive p potential in the biomedical domain. The self-assembled DNA materials derived from this technology are distinguished by their exceptional design flexibility, programmability, surface customizability, and favorable biocompatibility. These attributes promise to address critical issues such as low drug solubility, inadequate targeting, and high biological toxicity.

Despite these advantages, the translation of DNA origami into clinical utility confronts significant obstacles. A comprehensive understanding of the in vivo properties of DNA origami carriers is imperative, encompassing pharmacokinetics, intracellular trafficking, distribution, and clearance mechanisms. The potential encapsulation of DNA origami structures by protein coronas or uptake by macrophages upon entering the bloodstream could compromise their efficacy. Strategies such as encapsulating DNA origami with cationic shells, including polyethylene glycol [191] and peptide-based coatings, have demonstrated enhanced stability in physiological environments and prolonged circulating half-life [192]. Bastings et al. have shown that larger, more compact DNA structures can improve cellular uptake efficiency [193], while Jiang et al. have highlighted the renal enrichment effect of DNA origami structures [194]. These findings offer valuable insights into the behavior of DNA origami both in vitro and in vivo settings.

Furthermore, although DNA is an endogenous biomolecule, it may trigger immune responses under certain circumstances, necessitating the meticulous sequence design of the DNA strands [195]. The large-scale production of high-purity DNA nanostructures is also a critical challenge for the development of DNA origami carriers intended for clinical use. In addressing the aforementioned issue, Praetorius and colleagues developed a biotechnological method for the large-scale synthesis of DNA short strands, significantly reducing the cost of producing DNA origami structures and accelerating their clinical application as delivery vehicles [196].

As our understanding of DNA origami technology, pharmacokinetics, in vivo metabolic pathways, and interactions with disease targets deepen, future treatments are anticipated to leverage personalized and tailored DNA origami carrier systems. The DNA origami field is currently experiencing rapid growth, poised to unlock significant potential in the biomedical arena in the years to come.

### 4.4. Three-Dimensional Printing (3DP) Technology

Three-dimensional printing (3DP) refers to the computer-aided fabrication of three-dimensional objects through a layer-by-layer printing and assembly process. In 1996, Michael J. Cima of the Massachusetts Institute of Technology (MIT) first reported the application of powder bed fusion 3DP technology in the pharmaceutical domain [197,198]. Subsequently, with the emergence of pharmaceutical companies dedicated to 3DP and the growth of the pharmaceutical 3DP industry, this technology has garnered increased attention. 3DP technology offers a more streamlined approach compared to conventional manufacturing techniques, capable of efficiently producing objects with intricate geometries or complex internal structures. Its high level of control and flexibility make it particularly suited for the manufacture of personalized and innovative pharmaceutical formulations. In recent years, the pharmaceutical industry has shown a keen interest in 3DP technology, driven by its digital and customizable production capabilities. After extensive technical validation, the FDA has granted approval for Spritam (levetiracetam), the inaugural 3D-printed medication developed by Aprecia for the management of epilepsy. This innovative approach to pharmaceutical customization, offering precise dosage and release profiles, enhances both the efficacy and safety of treatment, with potential benefits extending to the management of psychiatric and neurological disorders. Wojtylko et al. have conducted a review of the application of 3D printing in solid dosage forms for the treatment of such conditions, highlighting its capacity to cater to the unique needs of pediatric patients and mitigate supply chain disruptions [199].

In addition, 3DP is making strides in various domains within the pharmaceutical sector. The straightforward degradation kinetics of biodegradable implants have led to the utilization of 3D printing for the generation of patient-specific implants. The employment of reductive polymerization techniques has facilitated the precision fabrication of small-scale medical devices, a practice currently applied in the manufacture of implants for the eye [200], cardiovascular system [201], and other anatomical locations. Studies have shown that 3D printing can simulate complex 3D microenvironments within the body, including the lung, gastrointestinal tract, and vascular system, as well as micro-robotic drug delivery systems. By utilizing materials such as hydrogels [202], poly(N-isopropylacrylamide) (PNIPAm) [203], 3D printing is also being adapted for drug delivery applications, addressing challenges related to resolution and scalability. As material manufacturing capabilities are integrated into 3D printing technology, the advent of 4D printing systems has emerged. This transformative technology has the capacity to dynamically respond to external stimuli such as temperature, light, or electric fields over time, enabling structural or functional self-transformation. This advancement has the potential to address the limitations of conventional therapies and to expand the frontiers of biomedical research through diverse and innovative applications [204].

Both the FDA and the Center for Drug Evaluation (CDE) of China have embraced a positive and inviting stance towards the integration of 3D printing (3DP) within the pharmaceutical sector. Nonetheless, the advancement and industrialization of 3DP in the realm of pharmaceuticals are confronted with considerable obstacles. Technological development demands rigorous research into the utilization of excipients in pharmaceutical procedures and in the design of drug formulations as well as in vivo and in vitro studies, the validation of release mechanisms, the elucidation of pharmacokinetics, and the assessment of the medicinal properties of novel three-dimensional structured drugs. Regarding the application of technology, it is imperative to refine the pertinent regulations concerning the continuous production technology of 3DP pharmaceuticals to guarantee the future commercialization of associated products.

Currently, digital and intelligent transformation stands as an essential pathway for the high-quality progression of the biopharmaceutical industry. As a critical component of this sector, the intelligent advancement of the pharmaceutical industry is non-negotiable. Lauded as the new “singularity” by the industry, 3DP drug technology represents a foundational revolution in pharmaceutical technology, offering a pivotal solution for the intelligent evolution of the pharmaceutical industry. Globally, the 3DP pharmaceutical industry is still in its nascent stages, and while the prospects for the development of 3DP drugs are promising, the process of its industrialization is prolonged and encumbered.

## 5. Extracorporeal Device

### 5.1. Microneedles

Microneedles (MN) represent an advanced transdermal drug delivery system, facilitating the passage of therapeutic agents of varying sizes into the skin. These devices can be designed to respond to certain endogenous or exogenous stimuli, such as pH value, reactive oxygen species (ROS), enzymes, light, temperature, and mechanical forces. This responsiveness allows for the controlled release of active compounds within the epidermis and dermis [205], thereby enhancing the stability of the drug and the delivery of various biomolecules [206]. Typically, microneedles are characterized by their height, which ranges from 10 to 2000 μm, and their width, which spans from 10 to 50 μm. Arrays of microneedles are designed to pierce the stratum corneum of the skin without contacting pain nerves, ensuring a painless and non-invasive delivery process that promotes both efficiency and patient compliance.

Microneedles are categorized into two types: solid and hollow. Solid microneedles are commonly fabricated from metallic materials or non-degradable polymers. For instance, Fitaihi et al. have developed an innovative ocular drug delivery system that utilizes a dissolvable MN array containing PLGA microparticles loaded with dexamethasone for scleral drug deposition [207]. This MN system demonstrated adequate mechanical strength to penetrate the porcine sclera. Additionally, He et al. [208] prepared two types of microneedles to enhance the in vitro transdermal delivery of progesterone drugs, thereby improving patient convenience and compliance. 

Hollow microneedles function as micro-sized injectors and have been effectively utilized for insulin delivery. The integration of microneedle arrays with nanosuspensions can augment the solubility of diclofenac [209]. The application of such nanomixed suspension microneedles to the skin has been documented to facilitate the delivery of diclofenac through the stratum corneum [210].

In addition, Yim et al. have developed grooved MNs that can be embedded into the skin through mechanical fracture under simple shear actuation. By fabricating an easy-to-operate applicator that provides adequate shear force, the tip of TCA-MN can be accurately delivered to the skin with a high probability (98% or more). The grooved MN platform has been proven capable of loading the required amount of drug and delivering it in the correct dosage [211]. A glucose-responsive “closed-loop” insulin delivery system that mimics the function of pancreatic cells has the potential to significantly improve the quality of life and health status of diabetic patients. Yu et al. [212] reported a novel glucose-responsive insulin delivery device using a painless microneedle-array patch (“smart insulin patch”). The smart insulin patch effectively regulated blood glucose in a chemically induced mouse model of type 1 diabetes. 

At present, microneedle transdermal drug delivery systems have been applied to small-molecule drugs, such as nicotine, painkillers, and therapeutic drugs for rheumatoid arthritis, neurological diseases, and tumors [213,214].

In recent years, microneedle transdermal drug delivery technology has been continuously iterated and developed, with the latest products featuring wearable and programmable capabilities. Currently, microneedle technology is highly integrated with electronic systems, flexible printed circuit board technology, microfluidic technology, glucose extraction, safe and sensitive continuous blood glucose monitoring, and other technologies. It has continuously made progress in intelligent, controllable, accurate and low reagent loss for drug release, and is being developed in conjunction with body fluid detection technology, wearable technology, macromolecule and targeted cellular drug delivery technology [215].

### 5.2. Needle Free Injection

Needle-free injection, also known as jet injection, was initially utilized for vaccine administration. This method employs a high-pressure jet generated by a pressure source to propel the drug through a thin nozzle, rapidly delivering the medication into the skin and facilitating its dispersion to the treatment site. Presently, this technique is utilized for the delivery of various drugs, such as insulin, lidocaine, DNA vaccines, and interfering RNA. A standard needle-free injection device operates by pressurizing liquids to approximately 20 MPa, with typical pressures ranging from 30 to 300 N. Additionally, it showcases the potential application of linear Lorentz-force motors in highly controllable, high-volume portable jet injectors, which can be beneficial for drug delivery in both animals and humans [216].

Needle-free injections are currently employed for administering medications such as liquid interferon, antibiotics, influenza vaccines, low molecular weight heparin, hepatitis B vaccines, insulin, morphine, lidocaine, erythropoietin, growth hormone, powder protein, peptide drugs, nucleic acid drugs, chemicals, and gene vaccines. This method is particularly favored by patients with phobias and pediatric patients, as it eliminates the risk of cross-infection associated with traditional syringe needles. However, it is important to note that the volume of medication that can be administered per needle-free injection is limited, and the injection must be administered perpendicular to the skin at the injection site to prevent increased pain and ensure complete penetration of the drug into the subcutaneous area. Due to the inability to accurately assess the depth of penetration, secondary supplemental drug administration is not feasible. As technology continues to advance, it is anticipated that these limitations will be addressed and resolved.

At present, needle-free drug delivery technology is advancing towards high-capacity delivery, self-care, and gene therapy. Needle-free thermoresponsive jet injectors have been shown to effectively deliver plasmid DNA to the skin, resulting in higher protein expression compared to needle injectors [217]. Large-capacity subcutaneous needle-free syringes have an injection tolerance of up to 2.0 mL, and the injection effect is generally better than two separate 1.0 mL injections using a needle-free injection system [218].

### 5.3. Micro Infusion Implantation Device

Intracranial local administration has been shown to reduce the risk of drug tolerance by connecting the implanted catheter to the subcutaneous drug reservoir or an external microinfusion pump with discontinuous/intermittent drug release through programmable microinfusion pumps. This method is primarily used to treat conditions such as epilepsy and refractory pain. A recent clinical study successfully utilized a subcutaneous implantable micro-infusion pump connected to a catheter system to deliver broad-spectrum valproic acid into the ventricles of patients with drug-resistant epilepsy [219]. The results showed effective improvement in the quality of life of the patient and no localized periventricular toxicity, although some mild adverse reactions were observed. Similarly, the intrathecal drug infusion system delivers drugs directly into the subarachnoid space, allowing the drugs to act directly on the spinal cord center for long-term pain control. This method is characterized by its good analgesic effect, with the dosage being only 1/1000 of intravenous medication and 1/300 of oral medication, significantly reducing the side effects of long-term medication. Once implanted, the operation is relatively simple and easy, significantly improving the quality of life of patients.

An innovative injectable capsule, known as L-SOMA, has been engineered

With an advanced driving and delivery mechanism. This system facilitates the encapsulation of both small-molecule drugs and large-molecule drugs, such as monoclonal antibodies, while doubling the drug loading capacity [220]. Remarkably, L-SOMA can achieve a peak plasma concentration akin to subcutaneous injection standards within a mere 30 min post-administration, and it can reach an impressive bioavailability of 80% within a few hours.

## 6. Challenges and Prospects

In the context of an aging global population and a surge in chronic diseases in both developed and developing nations, the demand for sophisticated drug delivery systems is escalating. These technologies have been pivotal in the development of numerous pharmaceutical products, particularly by enhancing the targeted delivery of therapeutic agents. A plethora of novel drug delivery technologies have emerged, focusing on maintaining drug stability, enhancing biocompatibility, and optimizing delivery pathways. For instance, a straightforward dehydration-rehydration method was employed to load the radiation protection drug amphotericin onto Spirulina, a natural and active microalgae. This approach resulted in an oral delivery system that offers comprehensive protection for the entire small intestine, prevents radiation-induced intestinal damage, preserves the stability of intestinal microbiota, and mitigates the long-term toxicity associated with amphotericin [221]. Similarly, the team of Zhou Min leveraged spiraled microalgae loaded with curcumin to facilitate the gradual release of the compound, thereby extending its retention time in the intestine and boosting the absorption efficiency and therapeutical efficacy of curcumin [222].

In the domain of device-based drug delivery, numerous innovative products such as microneedle and needle-free transdermal systems have been introduced internationally. Roughly 30 pharmaceuticals employing polymer and liposome delivery technologies and approximately 15 drugs utilizing antibody–drug conjugates (ADCs) have gained global authorization. In addition, around 40 medications based on extracellular vesicle delivery are currently in clinical trials, while biomimetic and live cell delivery systems are still in the research and development phase [223]. The international delivery systems sector encompasses a broader array of pioneering enterprises and demonstrates a more robust impetus for the advancement of drug delivery systems. Major corporate entities have escalated their investments in the drug delivery system market. Nonetheless, it is imperative to intensify foundational research into the technologies associated with novel delivery systems and to enhance our intrinsic innovative capabilities. In the realm of pharmaceutical advancement, drug delivery systems have evolved to encompass a spectrum of modalities, including macromolecular structures, cellular entities, sophisticated intelligent devices, and nanorobotic constructs, all of which serve as potential vectors for drug administration. The technologies and instruments integrated within the domain of delivery systems are both rich and varied, characterized by formidable technical challenges and a rapid pace of innovation. A myriad of drug delivery systems exists, each with its own set of merits and limitations, succinctly summarized in Table 3. Present clinical applications suggest that the integration of multiple technologies is the trajectory of future development. The synergistic employment of various drug delivery systems can mitigate their individual drawbacks and amplify their respective strengths. The quest for optimal delivery technologies in the pharmaceutical sector is unending, with ceaseless endeavors to refine and enhance drug delivery systems for greater efficacy. From the standpoint of veterinary practitioners, the application of drug delivery systems in the veterinary sphere remains relatively constrained.

From the standpoint of veterinary practitioners, the application of DDS in the veterinary sphere remains relatively constrained. Currently, sustained-release veterinary pharmaceuticals are predominantly antiparasitic and antibiotic agents, with oral and injectable forms being the primary dosage mechanisms. Targeted nanocarriers, particularly liposomes, are frequently utilized as drug delivery systems in veterinary medicine, while other nanomaterials are more commonly employed as feed supplements and have not been extensively investigated. Biological adhesive agents are predominantly utilized abroad for sedation and antibiotic delivery, and domestically for anti-inflammatory purposes [224]. Transdermal formulations are predominantly used abroad for analgesia and insecticide applications, and domestically as anti-inflammatory and antipyretic analgesics. Implantable controlled-release veterinary drug formulations are primarily used abroad for reproductive regulation and insulin-like sustained release, and domestically as anti-fertility and antiparasitic implants.

From the perspective of scientific researchers, drawing on the rapid development of drug delivery systems, the core of industrializing delivery system technology includes basic research, interdisciplinary research, and medical industry integration. Therefore, in future research, it is crucial to maintain a keen sense of industry hotspots, an open mindset towards technological collisions, and the principles of keeping up with the times and adhering to innovation.

## 7. Conclusions

Over the past two decades, drug delivery systems (DDSs) have undergone a remarkable transformation, transitioning from macroscale to nanoscale technologies and advancing towards intelligent, targeted delivery mechanisms. This paper presents a comprehensive review of the latest advancements in DDS technologies. Notably, nanoscale drug delivery systems have demonstrated remarkable flexibility by utilizing a diverse array of materials, including organic, inorganic, and hybrid organic-inorganic substances. These systems leverage the unique properties of nanoparticles, such as the small size effect, volume effect, surface effect, and quantum effect, to significantly enhance drug solubility, stability, and targeting capabilities. Consequently, they hold immense potential for applications within the biomedical field. However, several challenges persist that require resolution. The intricate interactions between nanocarriers and biological membranes, as well as the extracellular matrix, demand further detailed investigation. Additionally, the cytotoxicity and immunogenicity of these systems necessitate comprehensive nanotoxicological studies to evaluate their pharmacokinetic and pharmacodynamic properties in animal models. Biological carriers, which are derived from endogenous substances and retain the structural and functional attributes of their biological sources, have the potential to minimize undesirable immune responses. By emulating the structures of highly infectious agents or pathogens and replicating their internal mechanisms or modes of action, these carriers can deliver drugs with pinpoint accuracy to their intended targets, making them a highly promising approach for targeted DDS. Furthermore, some experts suggest that integrating DDS with cutting-edge technologies such as microfluidics, 3D printing, CRISPR-Cas9, and quantum sensing may represent the future of drug delivery systems. However, these innovative and creative approaches remain theoretical, and there are limitations to DDS approved by the FDA (Table 4). Therefore, extensive research and clinical trials are necessary to enhance the efficacy of these modern drug delivery systems for improved clinical applications. The synergistic use of multiple DDS is also a promising strategy, considering factors such as drug toxicity, adverse effects, mode of administration, and dosage. For instance, intelligent drug delivery systems based on nanocarriers can dynamically regulate drug release in response to changes in the tumor microenvironment, thereby enhancing therapeutic efficacy and reducing toxic side effects. The synergy of multiple DDS may lead to the development of new strategies with broader clinical applications and prospects compared to using a single drug delivery system. 

## Figures and Tables

**Table 1:** The challenges and solutions to drug molecular delivery.

Drug Molecular	Challenges	Solutions
Protac (Proteolysis Targeting Chimeras)	High molecular weight, poor bioavailability, poor stability	Antibody drug conjugates (ADC) Three-dimensional printing(3DP) Transdermal preparation Implanted catheter
Peptides and Proteins	Immunogenicity, short half-life,	Polymeric nanoparticles (PNPs) Peptide drug conjugate (PDC) Implanted catheter
Anti-body	Toxicity, Immunogenicity,	Cell drug delivery systems Antibody drug conjugates (ADC) Implanted catheter
Nucleic acid	Extrahepatic delivery, Immunogenicity, Enzyme degradation,	Liposomal drug delivery systems Viral drug delivery systems Bioparticle drug delivery systems Coupling targeted drug delivery systems
Cell	Unstable drug characteristics, Poor tissue permeability	Liposomal drug delivery systems Polymeric nanoparticles (PNPs)

**Table 2:** FDA approved ADCs.

Generic Name	Trade Name	Target	Payload/Payload Class	Payload Action	Approval Year
Mirvetuximab soravtansine	ELAHERE	FRα	Maytansinoid DM4	Folate receptor alpha	2022
Tisotumab vedotin-tftv	Tivdak	Tissue factor	MMAE/auristatin	microtubule inhibitor	2021
Loncastuximab tesirine-lpyl	Zynlonta	CD19	SG3199/PBD dimer	DNA cleavage	2021
Belantamab mafodotin-blmf	Blenrep	BCMA	MMAF/auristatin	microtubule inhibitor	2020, withdrawn in 2022
Sacituzumab govitecan	Trodelvy	TROP2	SN-38/camptothecin	TOP1 inhibitor	2020
Trastuzumab deruxtecan	Enhertu	HER2	DXd/camptothecin	TOP1 inhibitor	2019
Enfortumab vedotin	Padcev	Nectin4	MMAE/auristatin	microtubule inhibitor	2019
Polatuzumab vedotin-piiq	Polivy	CD79	MMAE/auristatin	microtubule inhibitor	2019
Moxetumomab pasudotox	Lumoxiti	CD22	PE38 (Pseudotox)	/	2018
Inotuzumab ozogamicin	Besponsa	CD22	ozogamicin/calicheamicin	DNA cleavage	2017
Trastuzumab emtansine	Kadcyla	HER2	DM1/maytansinoid	microtubule inhibitor	2013
Brentuximab vedotin	Adcetris	CD30	MMAE/auristatin	microtubule inhibitor	2011
Gemtuzumab ozogamicin	Mylotarg	CD33	ozogamicin/calicheamicin	DNA cleavage	2017; 2000

**Table 3:** Advantages and disadvantages of main novel drug molecular delivery systems.

DDS	Drug	Advantages	Disadvantages
Liposomal DDS	protac Peptides and proteins nucleic acid cell	low toxicity biocompatibility non-immunogenicity	low drug loading poor stability high production costs potential toxic side effects
Nanoparticles-based DDS	protac Peptides and proteins cell	biodegradability biocompatibility low toxicity safety and efficacy	potential toxicity unclear mechanism and polymer stability
Polymer Micelle DDS	insoluble protac Chinese herbal monomer	stability Solubilization low toxicity	long-term safety limitations in clinical application
Hydrogel DDS	protac Peptides and proteins	biocompatibility Biodegradability low toxic side effects	heavily depends on the internal microenvironment
Inorganic Nanoparticles DDS	protac Peptides and proteins nucleic acid	bioavailability low toxic side effects tolerance	unclear toxicity biological distribution, and clearance methods
cell DDS	anti-body protac Peptides and proteins nucleic acid Bacteria and viruses	biocompatibility low toxicity biological functions targeting low immunogenicity	poor release control limited loading capacity
Extracellular Vesicle DDS	Proteins, lipids, nucleic acids, sugars, and other macromolecules	cycling stability biocompatibility biological barrier permeability	immature technology unclear side effects
Viral DDS	nucleic acid	high infection rate, targeting, and mature technology	one time delivery high immunogenicity safety issues limited loading capacity
Bacterial DDS	protac Peptides and proteins	targeting good transport ability	weak survivability imprecise colonization safety issues
Bioparticle DDS	nucleic acid	targeting carry mRNA	unknown half-life and pharmacokinetics potential immunogenicity
Coupling Targeted DDS	protac anti-body nucleic acid Peptides and proteins	targeting extrahepatic delivery biodegradable low immunogenicity solubilization organizational permeability	enzymatic degradation chemical instability poor cycle stability immunogenicity high production cost
Intelligent DDS	protac Peptides and proteins nucleic acid cell	precise control targeting penetrate tissues structural designability programmability biocompatibility	unclear harmacokinetics, intracellular metabolic pathways, in vivo distribution, and clearance mechanisms
Extracorporeal Device	protac Peptides and proteins anti-body nucleic acid cell	Targeting improve drug delivery efficiency and patient compliance	developing towards high-capacity drug delivery

**Table 4:** FDA-approved DDSs and the signature drugs.

DDS	Trade Name	Significance	Year
the spansule technology	Spansule^®^	the first 12-h release technology	1952
	Contac^®^	delivering phenylpropanolamine hydrochloride and chlorpheniramine maleate	1974
	Dexedrine^®^	delivering dextroamphetamine sulfate	1982
polymer	Lupron Depot^®^	the first long-acting injectable PLGA polymer formulation	1989
	Abraxane^®^	drug-polymer composite nanoparticles	2005
liposomes	Doxil^®^	the first PEGylated liposome	1995
	Onpattro^®^	lipid-based nanoparticles used for the delivery of siRNA	2018
	Comiranty^®^	the first lipid-based nanoparticles used in COVID-19 vaccine	2021
nanomedicine	Rapamune^®^	the first nanocrystal formulation	2000
	Onpattro^®^	lipid-based nanoparticles used for the delivery of siRNA	2018
ADC	Mylotarg^®^	the first antibody–drug conjugate approved for clinical use	2009

## Data Availability

Not applicable.
